# Eltrombopag for Low-Risk Myelodysplastic Syndromes With Thrombocytopenia: Interim Results of a Phase II, Randomized, Placebo-Controlled Clinical Trial (EQOL-MDS)

**DOI:** 10.1200/JCO.22.02699

**Published:** 2023-06-09

**Authors:** Esther Natalie Oliva, Marta Riva, Pasquale Niscola, Valeria Santini, Massimo Breccia, Valentina Giai, Antonella Poloni, Andrea Patriarca, Elena Crisà, Isabella Capodanno, Prassede Salutari, Gianluigi Reda, Nicola Cascavilla, Dario Ferrero, Attilio Guarini, Giovanni Tripepi, Giuseppe Iannì, Emilio Russo, Andrea Castelli, Bruno Fattizzo, Germana Beltrami, Monica Bocchia, Alfredo Molteni, Pierre Fenaux, Ulrich Germing, Alessandra Ricco, Giuseppe A. Palumbo, Stefana Impera, Nicola Di Renzo, Flavia Rivellini, Francesco Buccisano, Aspasia Stamatoullas-Bastard, Anna Marina Liberati, Anna Candoni, Ilaria Maria Delfino, Maria Teresa Arcadi, Patrizia Cufari, Lorenzo Rizzo, Irene Bova, Maria Grazia D'Errigo, Gina Zini, Roberto Latagliata

**Affiliations:** ^1^ U.O.C. Ematologia, Grande Ospedale Metropolitano Bianchi Melacrino Morelli, Reggio di Calabria, Italy; ^2^ Dipartimento di Ematologia, Ospedale Niguarda Ca' Granda, Milano, Italy; ^3^ U.O. di Ematologia, Ospedale Sant'Eugenio, Roma, Italy; ^4^ U.O. di Ematologia, Azienda Ospedaliera Universitaria Careggi, Firenze, Italy; ^5^ Dipartimento di Ematologia Policlinico Umberto I, Università La Sapienza, Roma, Italy; ^6^ S.C. a Direzione Universitaria di Ematologia A.O., SS. Antonio e Biagio e Cesare Arrigo Alessandria, Alessandria, Italy; ^7^ Clinica di Ematologia Azienda Ospedaliera Universitaria—Ospedali Riuniti di Ancona, Ancona, Italy; ^8^ AOU Maggiore della Carità, Novara, Italy; ^9^ Candiolo Cancer Institute, FPO-IRCCS, Candiolo, Turin, Italy; ^10^ U.O. di Ematologia, A.U.S.L.-IRCCS di Reggio Emilia, Reggio Emilia, Italy; ^11^ Dipartimento Oncologico-Ematologico, Ospedale Civile Spirito Santo, Pescara, Italy; ^12^ Fondazione IRCCS Ca' Granda Ospedale Maggiore Policlinico, Milan, Italy; ^13^ U.O. Ematologia Ospedale Casa Sollievo della Sofferenza, San Giovanni Rotondo, Italy; ^14^ Dipartimento Biotecnologie Molecolari, Ematologia Universitaria A.O.U. Citta' della Salute e della Scienza di Torino, Turin, Italy; ^15^ U.O. Ematologia I.R.C.C.S. Istituto Tumori “Giovanni Paolo II”, Bari, Italy; ^16^ IFC-CNR Institute of Clinical Physiology, Reggio Calabria, Italy; ^17^ Dielnet SRL—CRO Reggio Calabria, Reggio Calabria, Italy; ^18^ Department of Pharmacology, University of Germaneto Catanzaro, Catanzaro, Italy; ^19^ SSD Ematologia Ospedale degli Infermi, Biella, Italy; ^20^ Dipartimento di Oncologia ed Emato-Oncologia, Università degli Studi di Milano, Milan, Italy; ^21^ U.O. Ematologia e terapie cellulari, IRCCS Azienda Ospedaliera Universitaria San Martino, Genova, Italy; ^22^ UOC Ematologia, Università di Siena, Azienda Ospedaliera Universitaria Senese, Siena, Italy; ^23^ Divisione di Ematologia, ASST di Cremona, Cremona, Italy; ^24^ Groupe Francais desmyélodysplasies (GFM), Paris, France; ^25^ Department of Hematology, Oncology, and Clinical Immunology, Heinrich-Heine-University Düsseldorf, Düsseldorf, Germany; ^26^ U.O. Ematologia con Trapianto, Azienda Ospedale Policlinicodi Bari, Bari, Italy; ^27^ Dipartimento di Scienze Mediche Chirurgiche e Tecnologie Avanzate “G.F. Ingrassia”, University of Catania, Catania, Italy; ^28^ U.O. C. Ematologia, A. O.ad Alta Specializzazione Ospedale Garibaldi Nesima, Catania, Italy; ^29^ U.O. di Ematologia, Ospedale Vito Fazzi, Lecce, Italy; ^30^ Divisione Ematologia, P.O. A. Tortora di Pagani-ASL Salerno, Pagani, Italy; ^31^ Divisione di Biopatologia e Diagnostica per Immagini, Policlinico Universitario Tor Vergata, Rome, Italy; ^32^ Centre Henri Becquerel, Rue d'Amiens, Rouen, France; ^33^ S.C. Oncoematologia, Università degli Studi di Perugia A.O. Santa Maria, Terni, Italy; ^34^ Divisione Ematologia, P.O. Santa Maria della Misericordia, A.S.U.F.C di Udine, Udine, Italy; ^35^ U.O. Farmacia Grande Ospedale Metropolitano Bianchi Melacrino Morelli, Reggio di Calabria, Italy; ^36^ Dipartimento di Ematologia, Ospedale Niguarda Ca' Granda, Milan, Italy; ^37^ U.O.S. di Genetica Medica Grande Ospedale Metropolitano Bianchi Melacrino Morelli, Reggio di Calabria, Italy; ^38^ Fondazione Policlinico, Universitario A. Gemelli IRCCS, Rome, Italy; ^39^ Università Cattolica del Sacro Cuore, Rome, Italy; ^40^ Divisione di Ematologia, Ospedale Belcolle, Viterbo, Italy

## Abstract

**PURPOSE:**

In myelodysplastic syndromes (MDS), severe thrombocytopenia is associated with poor prognosis. This multicenter trial presents the second-part long-term efficacy and safety results of eltrombopag in patients with low-risk MDS and severe thrombocytopenia.

**METHODS:**

In this single-blind, randomized, placebo-controlled, phase-II trial of adult patients with International Prognostic Scoring System low- or intermediate-1-risk MDS, patients with a stable platelet (PLT) count (<30 × 10^3^/mm^3^) received eltrombopag or placebo until disease progression. Primary end points were duration of PLT response (PLT-R; calculated from the time of PLT-R to date of loss of PLT-R, defined as bleeding/PLT count <30 × 10^3^/mm^3^ or last date in observation) and long-term safety and tolerability. Secondary end points included incidence and severity of bleeding, PLT transfusions, quality of life, leukemia-free survival, progression-free survival, overall survival and pharmacokinetics.

**RESULTS:**

From 2011 to 2021, of 325 patients screened, 169 patients were randomly assigned oral eltrombopag (N = 112) or placebo (N = 57) at a starting dose of 50 mg once daily to maximum of 300 mg. PLT-R, with 25-week follow-up (IQR, 14-68) occurred in 47/111 (42.3%) eltrombopag patients versus 6/54 (11.1%) in placebo (odds ratio, 5.9; 95% CI, 2.3 to 14.9; *P* < .001). In eltrombopag patients, 12/47 (25.5%) lost the PLT-R, with cumulative thrombocytopenia relapse-free survival at 60 months of 63.6% (95% CI, 46.0 to 81.2). Clinically significant bleeding (WHO bleeding score ≥ 2) occurred less frequently in the eltrombopag arm than in the placebo group (incidence rate ratio, 0.54; 95% CI, 0.38 to 0.75; *P* = .0002). Although no difference in the frequency of grade 1-2 adverse events (AEs) was observed, a higher proportion of eltrombopag patients experienced grade 3-4 AEs (χ^2^ = 9.5, *P* = .002). AML evolution and/or disease progression occurred in 17% (for both) of eltrombopag and placebo patients with no difference in survival times.

**CONCLUSION:**

Eltrombopag was effective and relatively safe in low-risk MDS with severe thrombocytopenia. This trial is registered with ClinicalTrials.gov identifier: NCT02912208 and EU Clinical Trials Register: EudraCT No. 2010-022890-33.

## INTRODUCTION

Myelodysplastic syndromes (MDS) are clonal disorders of the hemopoietic stem cell characterized by dysplastic hemopoiesis, cytopenias, and increased risk of evolution in AML.^1^

**CONTEXT**

**Key Objective**
Treatment of severe thrombocytopenia in patients with low-risk myelodysplastic syndromes (MDS) is a serious unmet need. Although we have previously documented the short-term efficacy and safety of the thrombopoietin receptor agonist eltrombopag in improving thrombocytopenia in low-risk MDS in the interim analysis of the first 50% of cases enrolled in the EQOL-MDS trial, we report on the second stage, which evaluates the long-term efficacy and safety of eltrombopag.
**Knowledge Generated**
In the full data set (N = 169) and longer follow-up (60 months), a high response rate is confirmed, which, in addition, is durable (60% still maintaining response at 5 years), and an improvement in other cytopenias was observed. Eltrombopag was observed to have an acceptable toxicity profile in the long term and is effective in raising and maintaining PLT counts at a safe level without any associated risk of MDS progression.
**Relevance *(C.F. Craddock)***
Administration of eltrombopag results in durable improvements in PLT counts in patients with low-risk MDS and appears to be well tolerated with acceptable toxicities to date. Longer-term follow-up of the trial cohort will be important.*
  *Relevance section written by *JCO* Associate Editor Charles F. Craddock, MD.

Although anemia prevails, severe thrombocytopenia (<30 × 10^3^/mm^3^ platelets [PLT]) is reported in at least 10% of subjects with lower MDS risk and has been recognized as an independent negative prognostic factor.^2-5^ Treatment is still generally limited to PLT transfusions^6^ since no effective drug is currently available for these patients, representing an unmet need in clinical practice.^7,8^

Thrombopoietin is the key regulator of PLT production by binding to its specific receptor thrombopoietin (TPO) receptor (TPO-R), on the megakaryocytic surface.^9^ A randomized, double-blind study with the TPO-R agonist, romiplostim, versus placebo for lower-risk MDS was stopped early because of an apparent increased risk of AML progression, which was not confirmed with long-term follow up.^10,11^

Eltrombopag is an orally bioavailable, small molecule acting as a TPO-R agonist, approved for the treatment of thrombocytopenia of chronic immune thrombocytopenic purpura, chronic hepatitis C virus infection, and for acquired severe aplastic anemia.^12,13^ In higher-risk MDS, the addition of eltrombopag to azacitidine resulted in worse PLT recovery and increased progression to AML.^14^

We have previously reported on the short-term outcome of the first 90 cases enrolled in the EQOL-MDS trial, a phase-II, randomized study designed to assess eltrombopag efficacy and safety compared with placebo in patients with lower-risk MDS with severe persistent thrombocytopenia.^15^ The primary end points of the first part of the trial demonstrated encouraging safety and superiority of eltrombopag in inducing PLT response (PLT-R) compared with placebo, making this drug a promising approach for the management of thrombocytopenia in low-risk MDS.^16^ We now report the predefined interim results on the entire sample enrolled in the EQOL-MDS trial with follow-up of at least 3 months (169 cases).

## METHODS

### Trial Design

EQOL-MDS is an international, multicenter, randomized, single-blind, placebo-controlled, superiority trial (additional information on trial design is provided in the Data Supplement [online only]).

The trial Protocol (online only) was approved by an independent ethics committee at each participating institution, and all patients provided written informed consent.

### Patients

Inclusion criteria were patients age 18 years and older with diagnosis of low or intermediate-1 International Prognostic Scoring System (IPSS)^2^ risk MDS with stable PLT count (<30 × 10^3^/mm^3^ without exceeding >200 Gi/L) confirmed by blinded central evaluation, and refractoriness or ineligibility to receive, or relapsed while receiving treatment with alternative medications.

Exclusion criteria were (1) prior chemoradiotherapy or previous treatment with TPO-R agonists; (2) peripheral monocytosis > ×10^3^/mm^3^ or leukocytosis ≥ ×10^3^/mm^3^; (3) marrow fibrosis with an inability to aspirate marrow; (4) Eastern Cooperative Oncology Group performance status^17^ >3; (5) serum creatinine >2 times the upper limit of normal (ULN), AST or ALT >3 times the ULN or bilirubin >1.5 times the ULN; and (6) pre-existing cardiovascular disease or arrhythmia associated with an increased risk of thromboembolic event. Cases with >5% bone marrow blasts were excluded in France. Erythropoiesis-stimulating agents or granulocyte colony-stimulating factor was permitted during the trial, as per accepted standards. Additional information is provided in the Data Supplement.

### Trial Procedures and Treatment

#### Random Assignment and Masking

Participants were randomly assigned (2:1) to either eltrombopag or matching placebo as previously described.^15^ Additional information is provided in the Data Supplement.

#### Laboratory Assessments

Peripheral blood and bone marrow assessments were performed during screening before random assignment and preselected time points throughout the trial (Data Supplement).

#### Eltrombopag Administration

Oral eltrombopag or matching placebo was administered at an initial dose of 50 mg once daily, titrated in 50-mg increments every 2 weeks up to 300 mg to achieve a complete PLT-R, defined as a PLT count ≥100 × 10^3^/mm^3^ without bleeding (Data Supplement).

#### Assessment of Quality of Life

Change in quality-of-life (QoL) scores were measured at baseline and at subsequent time points using the European Organisation for Research and Treatment of Cancer Quality of Life Questionnaire C30 questionnaire^18^ and MDS-specific QOL-E questionnaire^19^ (Data Supplement).

#### Eltrombopag Pharmacokinetic Analysis

Data on the methodology used to process plasma samples for eltrombopag pharmacokinetic analysis are provided in the Data Supplement.

#### Study End Points

The first part of the trial determined short-term efficacy and safety at 50% accrual, as previously described^15^; the second part, reported in the present predefined interim analysis, evaluates the long-term response and safety (Data Supplement [Fig S1]). A PLT-R was defined as achieving the following increases in PLT count from baseline levels: for patients with baseline PLTs at least 20 × 10^3^/mm^3^, an increase of at least 30 × 10^3^/mm^3^ from baseline; and for patients with baseline PLTs <20 × 10^3^/mm^3^, an increase of more than 20 × 10^3^/mm^3^ and an increase of at least 100%, not because of PLT transfusions, in the absence of bleeding (Data Supplement).

Part 2 primary end points include duration of PLT-R and long-term safety and tolerability. Secondary end points include (1) difference in time to response (time from starting treatment to time of achievement PLT-R); (2) frequency of PLT transfusions during the treatment and follow-up periods; (3) duration of PLT transfusion independence; (4) incidence and severity of bleeding using the WHO Bleeding Scale^20^; (5) progression-free survival (PFS), leukemia-free survival (LFS), and overall survival (OS); (6) changes in QoL scores; and (7) eltrombopag population pharmacokinetic parameters and plasma concentration data.

### Statistical Analysis

Data are summarized as mean ± standard deviation (normally distributed variables), median and IQR (non-normally distributed variables), or as percent frequency (binary/categorical variables), and between-arms comparisons were performed by independent *t* test, Mann-Whitney *U* test, or Pearson's chi-square test, as appropriate.

Efficacy and safety analyses were performed on the full analysis set, that is, randomized patients who had received at least one dose of eltrombopag, according to the modified intention-to-treat (ITT) principle.

The primary end point (PLT-R) of the 24-week trial (first part) was analyzed with the use of a logistic regression model that included the trial group as an independent variable and was also presented graphically by reverse Kaplan-Meier curves and compared by log-rank test. PLT-R, defined according to International Working Group 2006 criteria,^21^ was assessed at each visit. A response (PLT-R) required an absence of bleeding, with the following increases in PLT count from baseline levels: for patients with baseline PLTs ≥20 × 10^3^/mm^3^, an increase of at least 30 × 10^3^/mm^3^ from baseline; for patients with baseline PLTs <20 × 10^3^/mm^3^, the achievement of >20 × 10^3^/mm^3^ and an increase of at least 100%, not because of PLT transfusions.

To account for differences in follow-up time between patients of the two study arms, we also performed a time-to-event analysis by a reverse Kaplan-Meier method.

The duration of PLT-R (primary end point of second part) was calculated from the time of PLT-R to the date of loss of PLT-R, defined as bleeding or PLT count <30 × 10^3^/mm^3^ or last date in observation for those who did not lose the response. The time to the loss of PLT-R was investigated by Kaplan-Meier curves.

Safety outcomes (primary end point of both parts) were summarized descriptively. Between-treatment comparisons (adverse events [AEs], progression of MDS, AML evolution, and death) were conducted using a chi-square test. Additional information is provided in the Data Supplement.

## RESULTS

### Patient Characteristics

**FIG 1.**
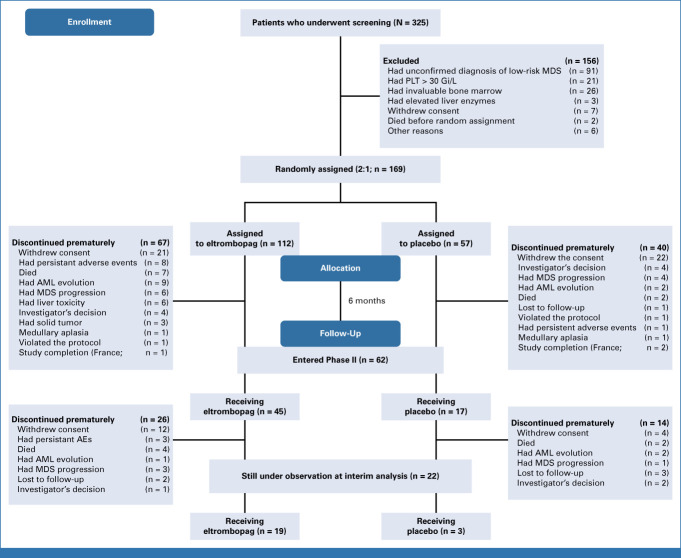
CONSORT diagram. Four patients (one in the eltrombopag arm and three in the control arm) withdrew from the trial before receiving the first dose of eltrombopag; thus, 165 patients (111 patients of the eltrombopag arm and 54 patients of the control arm) were considered in the modified ITT. The first patient was enrolled on June 13, 2011, and the last patient was enrolled on October 1, 2021. At the time of data extraction, 45 patients of 111 of the eltrombopag group and 17 patients of 54 of the placebo group entered the second part of the trial. AE, adverse event; ITT, intention-to-treat; MDS, myelodysplastic syndrome; PLT, platelet.

At data cutoff, March 3, 2022, of the 325 patients screened, 169 patients were randomly assigned eltrombopag (N = 112) or placebo (N = 57; Fig 1 and Table 1). Of these, 165 patients received at least one dose of study drug (111 in the eltrombopag arm and 54 in the placebo arm) and were considered in the modified ITT analysis. Baseline features were similar in the two arms (Table 1). Main MDS-related concomitant treatments were erythropoiesis-stimulating agents, steroids (16.0% for both), and deferasirox (5.9%).

**TABLE 1.**
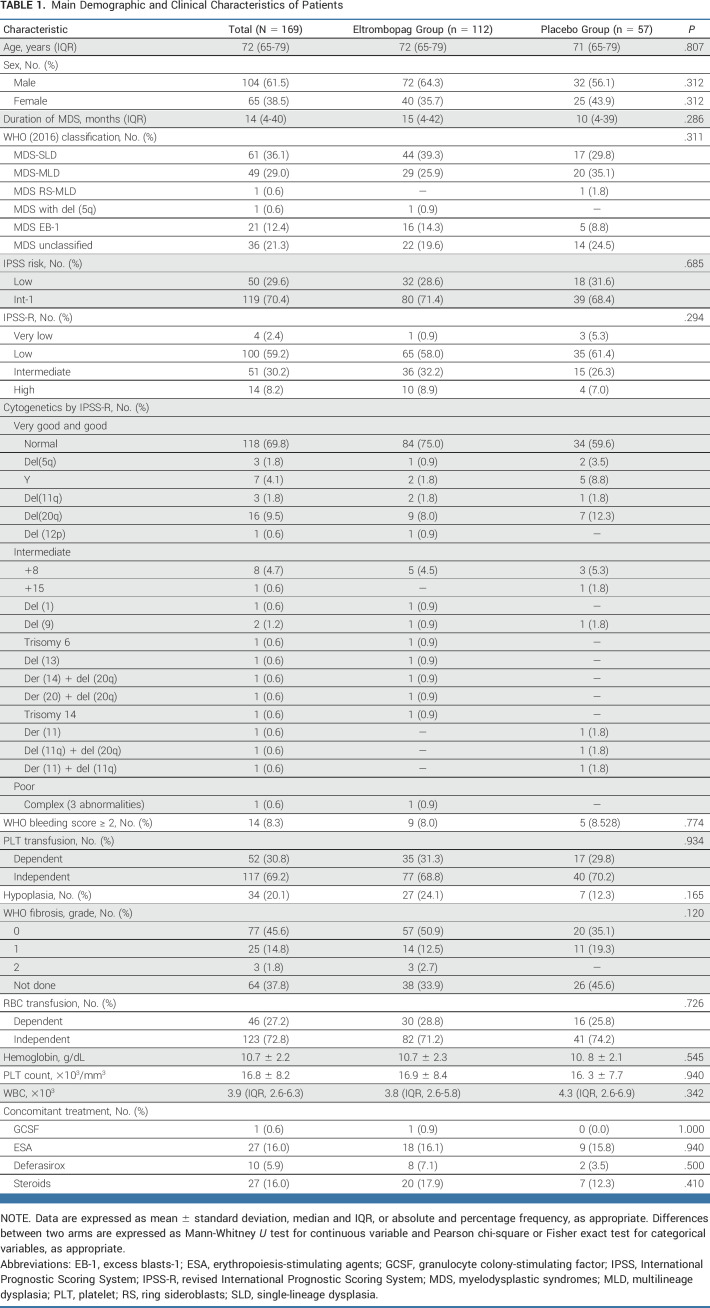
Main Demographic and Clinical Characteristics of Patients

### Primary End Points: Efficacy and Safety

#### PLT Response

**FIG 2.**
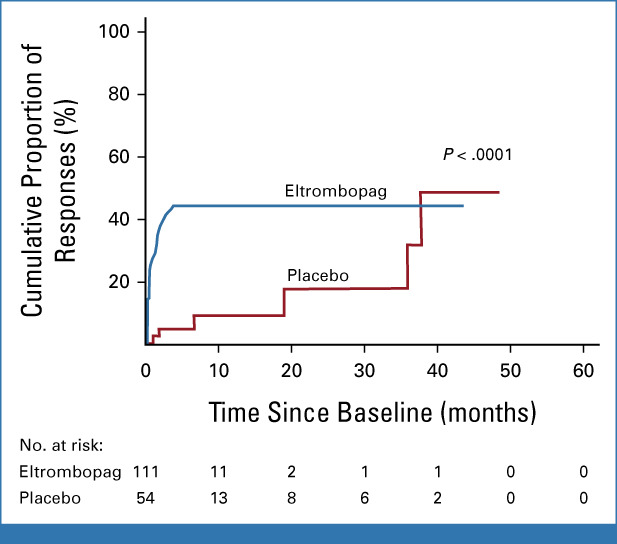
Reverse Kaplan-Meier curves of platelet response in both treatment groups.

The median follow-up time in all patients was 25 (IQR, 14-68 weeks); 27 (IQR, 15-71 weeks) in the eltrombopag arm and 22 (IQR, 12-52 weeks) in the placebo arm. PLT-R was observed in 47 (42.3%) eltrombopag recipients, of whom 31 (66.0%) showed a complete response, compared with six (11.1%; none were complete) in 54 placebo recipients (odds ratio, 5.9; 95% CI, 2.3 to 14.9; *P* < .001; Fig 2).

**FIG 3.**
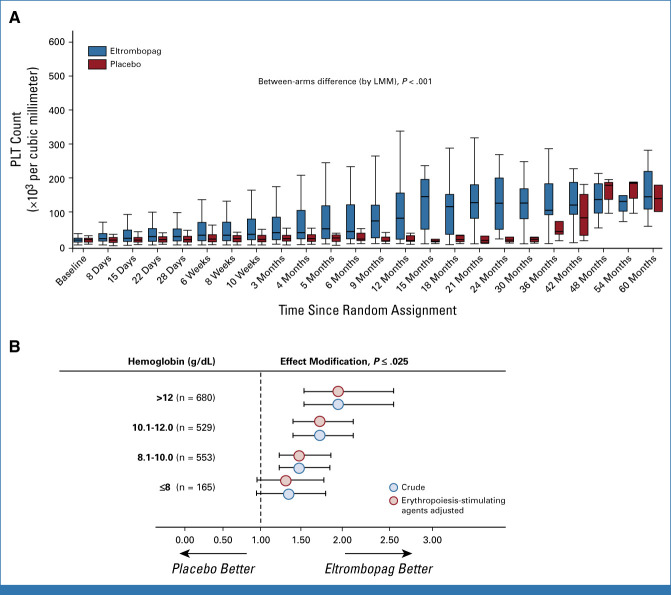
(A) Box and whisker plot of PLT count over time. The horizontal line in the middle of each box represents the median, while the top and bottom of each box represent the 75th and 25th percentiles, respectively. The whiskers above and below the box plot mark the 97.5th and 2.5th percentiles, respectively. (B) Hemoglobin as an effect modifier of PLT response (log-transformed data) to eltrombopag. Data represent the ratio (and 95% CI) over the study period of the geometric mean of PLT count in eltrombopag-treated patients to the geometric mean of PLT count in patients in the placebo group. The numbers reported in parentheses (B) represent the number of observations with a hemoglobin value over time falling in a specific hemoglobin interval. LMM, linear mixed models; PLT, platelet.

The median daily drug dose at response was 50 (IQR, 50-150 mg). All responsive patients required dose titration during the first 6 months. Each titrated dose over the whole study period is summarized in the Data Supplement (Table S1). Median PLT increment at best response in the first 24 weeks was significantly higher in the eltrombopag (31; IQR, 8-103 × 10^3^/mm^3^) compared with the placebo arm (9.5 [IQR, 1.75-17] × 10^3^/mm^3^; *P* < .0001; Fig 3A).

In a linear-mixed model, hemoglobin was found to modify the PLT-R to eltrombopag, with the between-treatment difference in PLT count over the trial period being closely related to hemoglobin levels (Fig 3B). The effect of eltrombopag became apparent at a hemoglobin level of 8.1 g/dL and increased linearly. No effect modification by MDS duration, WHO classification, IPSS,^2^ revised IPSS,^3^ cytogenetics, bleeding, fibrosis, and hypoplasia was found on PLT-R to eltrombopag. In a multivariate Cox model, only baseline hemoglobin levels (hazard ratio [1 g/dL], 1.21; 95% CI, 1.07 to 1.38; *P* = .003) maintained a significant association with PLT-R. Additional analysis is provided in the Data Supplement.

#### Duration of PLT-R

At the time of data cutoff, 16 of 47 (34.0%) responsive patients in the eltrombopag arm are still responsive on treatment versus one of six (16.6%) responsive placebo patients. Median duration of response in the whole group was not reached during the entire observation period. In the eltrombopag arm (observation period of 13-561 weeks), 12 of 47 (25.5%) of eltrombopag subjects lost the PLT-R with a cumulative thrombocytopenia relapse-free survival at 60 months of 63.6% (95% CI, 46.0 to 81.2; Data Supplement [Fig S2]). Among 12 patients who lost PLT-R, one patient recovered a response by increasing eltrombopag from 100 mg to 200 mg. The remaining 19 eltrombopag responders terminated the study still in PLT-R for various reasons. No difference in PLT-R duration was observed between responders of the active arm and those of the control arm (log-rank test = 2.16; *P* = .14).

In the placebo arm (observation period of 83-398 weeks), the six placebo responders maintained the PLT-R until study termination. One remains in the study after 76 months of response.

Additional results on PLT-R and reasons for early study termination are provided in the Data Supplement.

#### Safety

At the time of data cutoff, 15 unrelated deaths occurred: 11 (9.9%) in the eltrombopag arm for infection in five, cardiorespiratory failure in two, hemorrhage in one, and worsening of general condition in three; and four (7.4%) in the placebo arm for infection, heart failure, hemorrhage, and worsening of general condition. Median survival was not reached for both arms with no difference in overall cumulative survival between arms (log-rank test; *P* = .502; Data Supplement [Fig S3]). Sixty-two patients in the eltrombopag arm (exposure adjusted incidence rate [EAIR], 3.1 events per 100 persons-month; 95% CI, 2.4 to 4.0) and 29 in the placebo arm (EAIR, 4.1; 95% CI, 2.8 to 5.9) experienced grade 1-2 AEs (EAIR, 0.76; 95% CI, 0.48 to 1.23; *P* = .23; Data Supplement [Table S2]) with no significant difference between arms (*P* = .868). Fifty patients in the eltrombopag arm (EAIR, 2.5; 95% CI, 1.9 to 3.4) and 11 in the placebo arm (EAIR, 1.6; 95% CI, 0.8 to 2.8) experienced grade 3-4 nonhematologic AEs (EAIR, 1.62; 95% CI, 0.83 to 3.46; *P* = .14; Data Supplement [Table S3]), with a significant difference between arms (*P* = .002) favoring placebo, but the stopping rule was not reached. Eighteen eltrombopag patients required permanent treatment discontinuation because of severe liver or persistent grade 3-4 AEs after a median time from baseline of 13 weeks (IQR, 6-20), whereas no placebo cases experienced such events.

### Secondary End Points

#### Frequency and Duration of PLT Transfusion Independency

Among PLT transfusion-dependent patients (Table 1), 19 eltrombopag cases of 35 (54.3%) achieved PLT transfusion independence and 12 cases were associated with a PLT-R.

In the control group, six of 17 cases (35.3%) achieved PLT transfusion independence with a PLT-R in two cases (between-arm difference; *P* = .24). Median duration of PLT transfusion independence was 32 weeks (IQR, 14-64) in eltrombopag and 9 weeks (IQR, 2-98) in the control arm (*P* = .15). The incidence rate of PLT transfusion was on average 16.3 per 100 patients-week (95% CI, 15.4 to 17.2) in the active arm and 16.9 per 100 patients-week (95% CI, 15.5 to 18.4) in the control arm (*P* = .49).

#### Median Time to PLT-R

The median time to PLT-R was significantly earlier in the eltrombopag arm (2.1 weeks, 1-6 weeks) versus the placebo arm (54 weeks, 6-155 weeks; *P* < .001).

#### Incidence and Severity of Bleeding

Thirty-nine subjects had at least one clinically significant bleeding event (WHO bleeding score ≥ 2) during the entire study period: 17 (31.5%) cases in the placebo group (exposure-adjusted event rate [EAER], 7.3 events per 100 patients/week) versus 22 eltrombopag cases (19.8%; EAER, 3.9 events per 100 patients/week; incidence rate ratio, 0.54; 95% CI, 0.38 to 0.75; *P* = .0002). The distribution of patients according to the maximum degree of bleeding is provided in Table 2.

**TABLE 2.**
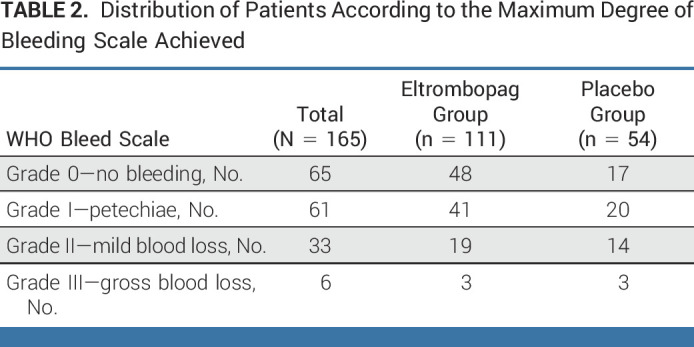
Distribution of Patients According to the Maximum Degree of Bleeding Scale Achieved

#### Progression of MDS, AML Evolution, LFS Survival, PFS, and OS

Progression of MDS occurred in nine eltrombopag (8%) versus five placebo subjects (9%; χ^2^ = 0.062; *P* = .774). AML evolution occurred in 10 of 111 (9%) eltrombopag patients after a median time from treatment start of 13 weeks (11-24) versus four of the 54 (7%) placebo patients (χ^2^ = 0.120; *P* = .729) after a median time from treatment start of 34 weeks (7-133). The low number of events precluded the possibility to investigate predictors of evolution to AML.

Overall, the combined outcome AML evolution and/or disease progression occurred in 19 of 111 (17%) eltrombopag patients versus nine of 54 (17%) placebo patients (χ^2^ = 0.005; *P* = 1.0). Progression was related to natural disease course of disease and was not transient. Eltrombopag was stopped after progression to MDS or AML. No transient increases in circulating peripheral myeloblasts were observed.

Five-year cumulative LFS, PFS, combined outcome-free survival (CFS), and OS in the eltrombopag arm were 65.0% (95% CI, 51.7 to 78.3), 61.4% (95% CI, 45.3 to 77.5), 54.0% (95% CI, 39.3 to 68.7), and 77.3% (95% CI, 64.4 to 90.2), respectively, compared with 44.9% (95% CI, 4.3 to 85.5), 44.6% (95% CI, 5.8 to 83.4), 34.5% (95% CI, 2.4 to 66.6), and 58.1% (95% CI, 9.7 to 100.0) in the placebo arm, and did not reach statistical significance. Additional results are available in the Data Supplement ([Figs S4 and S5]). The number needed to treat for a PLT-R of at least 4 weeks' duration was 3.2, and the number needed to harm for grade 3-4 AEs was 4.1. The number needed to observe AML evolution was 62. Thus, the likelihood of being helped or harmed, in terms of serious AEs versus efficacy, is 1.3 and AML evolution versus efficacy is 19.4. Therefore, eltrombopag treatment is 1.3-19.4 times more likely to help in terms of PLT-R than to harm.

#### Other Secondary Outcomes

Patient-reported outcomes (Data Supplement [Tables S4-S7]) and pharmacokinetic analysis (Data Supplement [Figs S6A, S6B, S7A, and S7B, and Table S8]) are presented in the Data Supplement. In the eltrombopag arm, 66 patients were anemic at baseline: 18 (27.3%) achieved a durable and significant erythroid improvement and 11 of 30 (36.7%) red blood cell transfusion-dependent patients became transfusion-independent. Only nine (50%) were PLT responders. In addition, 27 patients in the eltrombopag arm had grade 3-4 neutropenia (<1.0 × 10^3^/mm^3^) at baseline. A durable neutrophil response was observed in five (18.5%) patients, of whom two were not PLT responders.

- Median LFS, combined outcome (AML, disease progression, and death), and OS were not reached in the whole group.

## DISCUSSION

The occurrence of severe thrombocytopenia in patients with low-risk MDS still represents a challenging condition because of the important burden of related AEs and the lack of effective treatments. In most countries, only PLT transfusions are currently offered to prevent or treat bleeding in these subjects. Despite this unmet clinical need, few investigational products have been tested. Among them, TPO mimetics are the most attractive: however, though the use of romiplostim seemed promising in a randomized trial,^10^ there is still concern regarding the risk of MDS progression/AML evolution and warning in the label of all TPO mimetics.^16^ Eltrombopag has a different mechanism of action from romiplostim. Furthermore, a trilineage marrow response is observed in severe aplastic anemia.^22^

Preliminary results of this trial already showed high rates of PLT-R in this challenging subset of patients.^15^ Our present predefined interim analysis report on the long-term efficacy and safety of eltrombopag. Our previous part-1 results included 90 patients with a follow-up of 6 months and a median follow-up of 11 weeks.^15^ The present part-2 interim results confirm our previous results in terms of obtaining a durable and stable PLT-R and extend them further where we included 169 randomly assigned patients with a follow-up of 60 months (a median follow-up of 25 weeks).

Importantly, the findings from the present trial show a favorable safety profile in terms of long-term AEs and AML evolution/disease progression.

While the rate of PLT-R was confirmed, a complete PLT-R (27.9%) was observed in the eltrombopag arm only. Occurrence of a small fraction of PLT-R in the placebo arm was probably because of the spontaneous variability of hematologic parameters in patients with MDS during the course of the disease, as predetermined by the sample size calculation and as commonly observed also in other randomized studies in patients with low-risk MDS.^23-25^ The achievement of PLT-R occurred significantly earlier in the eltrombopag patients with a higher median PLT count increment in responders compared with placebo responsive patients (*P* < .001). PLT-R to eltrombopag was durable, with 60% of patients still maintaining response at 5 years. Moreover, although only a fraction of patients were receiving platelet transfusions at study entry, the rate of patients achieving PLT transfusion independence was higher and, importantly, the rate of incidence of bleeding was lower in the eltrombopag arm than in the placebo arm, reaching statistical significance.

Our findings also corroborate with results from a recent multicenter retrospective study performed in France to evaluate the use of eltrombopag in patients with MDS (N = 50) and chronic myelomonocytic leukemia (N = 11).^26^ PLT-R occurred in 47 (77%) patients, a higher rate than what we observed (42.3%), possibly attributed to differences in baseline clinical characteristics.

The dose of eltrombopag (50 mg once daily) to obtain PLT-R was the same as that used in our trial but we observed a substantially shorter median time to reach PLT-R (2.1 *v* 4.3 weeks) that may be due to the higher frequency of blood counts undertaken in our trial. Disease progression was observed in 16% of patients, almost identical to the rate that we observed in both eltrombopag and placebo arms (17%).

Similar to what is observed in aplastic anemia, a sizable fraction of patients receiving eltrombopag also achieved an erythroid/granulocytic response. This particular finding deserves further evaluation in larger cohorts of cytopenic patients with low-risk MDS.

As expected, patients in the eltrombopag arm had a higher rate of nonhematologic AEs and 18 patients necessitated permanent treatment discontinuation because of persistent drug-related AEs. Most grade 3-4 AEs occurred early during treatment, within the first 24 weeks, and were reversible upon drug discontinuation. However, no differences were reported between the two arms for deaths.

The most important safety evaluation, however, concerned the risk of the increase in blasts and MDS progression/AML evolution, which was shown during romiplostim treatment in a similar trial.^10^ In the present trial, long-term data are encouraging, with no transient increase in circulating peripheral myeloblasts and similar 5-year LFS, PFS, and CFS in the two arms.

Finally, it should be stressed that a relatively low daily dose of eltrombopag (50 mg once daily) was identified as the optimal dose at which patients more commonly achieved a consistent PLT-R, a finding also confirmed in the real-life setting,^26^ with a lower risk of AEs compared with higher doses. Besides the important clinical benefit in terms of efficacy with associated low risk of AEs over the long term, the use of low-dose eltrombopag may also translate into significant reduction in health care costs. Although there is evidence of an immunomodulatory role and the ability to mobilize intracellular iron,^27,28^ the biological mechanisms between eltrombopag and the immune system still need to be elucidated, particularly at low doses. On the basis of our long-term results, we may conclude that there is no major advantage in a dose increase beyond 150 mg, limited to selected cases at high risk of bleeding.

In conclusion, eltrombopag has an acceptable toxicity profile and is effective in raising and maintaining PLT count and reducing bleeding without any associated risk of MDS progression.
